# CircTENM3 inhibites tumor progression via the miR-558/RUNX3 axis in prostate cancer

**DOI:** 10.1186/s12967-023-04708-0

**Published:** 2023-11-25

**Authors:** Lingxiang Lu, Fei Wang, Jianchun Chen, Chunchun Zhao, Shuai Guo, Da Dong, Minjun Jiang, Yuhua Huang

**Affiliations:** 1 Department of Urinary Surgery, Suzhou Ninth People’s Hospital, Soochow University, Suzhou, Jiangsu China; 2 Department of Urology, The Affiliated Suzhou Hospital of Nanjing Medical University, Suzhou Municipal Hospital, Gusu School, Nanjing Medical University, Suzhou, Jiangsu China; 3 Department of Urinary Surgery, The First Affiliated Hospital of Soochow University, Suzhou, Jiangsu China; 4 Suzhou Ninth People’s Hospital, Soochow University, Suzhou, Jiangsu China

**Keywords:** PCa, circTENM3, miR-558, RUNX3

## Abstract

**Background:**

Prostate cancer (PCa) is currently acknowledged as the second most widespread cancer among men worldwide. Yet, the lack of dependable diagnostic biomarkers and therapeutic targets has presented considerable hurdles to the progression of prostate cancer treatment. Circular RNAs are implicated in the pathogenesis of numerous diseases, positioning them as promising biomarkers for diverse medical conditions. This study aims to uncover a specific circRNA that could serve as a diagnostic and therapeutic target for detecting and treating PCa.

**Methods:**

The change of circTENM3 expression levels in PCa was detected by qPCR. CCK8 assays, EdU assays, Scratch assay and Transwell migration assay conducted to detect the role of circTENM3 in PCa cells in vitro. RIP assay, RNA-pull down and luciferase reporter assay were performed to explore the mechanism of circTENM3. Gain-of-function analysis was performed to reveal the function of circTENM3 in PCa in vivo.

**Results:**

The results revealed that the expression level of circTENM3 was significantly down-regulated in PCa. CircTENM3 overexpression alleviated the progression of PCa in vitro. Mechanistically, circTENM3 enhanced RUNX3 levels via miR-558 sponge. Gain-of-function analysis determined that circTENM3 overexpression could inhibit PCa progression in vitro.

**Conclusions:**

Our research offers profound insights into the protective role played by circTENM3 in PCa. CircTENM3 operates as a sponge for miR-558, thereby triggering the elevation of RUNX3 expression, which subsequently curbs the progression of PCa.

**Supplementary Information:**

The online version contains supplementary material available at 10.1186/s12967-023-04708-0.

## Introduction

Prostate cancer (PCa) is presently recognized as the second most prevalent malignancy in men on a global scale [1, 2]. The widespread adoption of prostate-specific antigen (PSA) screening has facilitated the detection of approximately 10 million cases of PCa among men, with an additional 1.3 million new cases being identified annually across the world [3, 4]. Despite these staggering figures, the absence of reliable diagnostic biomarkers and therapeutic targets has posed significant challenges to the advancement of PCa treatment [5, 6]. Hence, the exploration for potent biomarkers or targets that can shed light on the underlying molecular mechanisms responsible for PCa progression has become an indispensable pursuit.

Circular RNA (circRNA) is a unique type of endogenous non-coding RNA characterized by its closed-loop structure, lacking the typical 5′ cap and 3′ poly(A) tail [7–9]. Due to this distinct feature, circRNA exhibits high resistance to degradation by exonucleases and digestion by RNase R [10]. Initially, circRNA received little attention due to its low expression levels. However, with the development of high-throughput sequencing technology and specialized bioinformatics algorithms for circRNA detection, circRNAs have gained significant recognition [11]. Notably, a single gene can generate multiple different circRNAs, surpassing the diversity of protein-coding genes in human cells [12]. Emerging evidence suggests that certain circRNAs are abnormally expressed in various tumors [13, 14]. Moreover, hundreds of circRNAs are associated with diverse physiological and pathological processes, such as tumor initiation, development, and immune evasion, indicating their potential functional significance [15, 16].

Emerging research has demonstrated that circRNAs possess the capacity to function as microRNA (miRNA) sponges or protein decoys, and in certain instances, they can encode small peptides, thus exerting significant biological functions [7, 17, 18]. Such actions can potentially disrupt target genes that are relevant to cancer biology. Notably, recent investigations have associated specific circRNAs with the malignancy-related behaviors of PCa cells, influencing critical processes such as proliferation, migration, invasion, and epithelial-mesenchymal transition [19–21]. Nevertheless, the comprehensive pathophysiological implications of the majority of differentially expressed circRNAs in PCa development remain largely unexplored.

Within the scope of our current investigation, we have successfully identified a novel circRNA, hsa_circ_0071478, derived from the TENM3 gene, exhibiting noteworthy upregulation in PCa. Notably, this circRNA demonstrates a strong correlation with the survival outcomes of PCa patients. Mechanistically, circTENM3 predominantly localizes in the cytoplasm and exerts inhibitory effects on the progression of PCa through the modulation of the miR-558/RUNX3 axis. These compelling findings offer valuable insights into the potential roles of circRNAs as promising biomarkers and therapeutic targets in the context of PCa.

## Methods

### Cell culture

PCa cell lines, namely PC3 and DU145, were cultivated in DMEM medium (Gibco, USA) at 37 °C with 5% CO_2_ and saturated humidity. The culture medium was supplemented with 10% fetal bovine serum (FBS, Gibco) and 1% penicillin–streptomycin (P/S, Gibco). For gene modulation, small interfering RNA (siRNA) and miRNA mimics were synthesized by GenePharma Co., Ltd. (Shanghai, China). Transient transfections were carried out using Lipofectamine 2000 (Invitrogen), following the manufacturer's instructions. GeneChem Co., Ltd. (Shanghai, China) was responsible for the design and construction of adenovirus expressing hsa_circ_0071478, as well as a negative control. The target sequences utilized are detailed in Additional file 1: Table S1.

### Quantitative polymerase chain reaction (qPCR)

Total RNA from both PCa tissues and cells was isolated using the TRIzol reagent (Invitrogen). Reverse transcription was conducted using the HiScript III RT SuperMix (Cat#R323, Vazyme), a high-quality reagent for converting RNA to cDNA. qPCR was performed with the ChamQ Universal SYBR qPCR Master Mix (Cat#Q711, Vazyme), following the manufacturer's guidelines. For normalization purposes, β-actin and U6 were employed as housekeeping controls for circRNA/mRNA and miRNA expression, respectively. All the miRNA primers were obtained from RiboBio Co., Ltd. (Guangzhou, China). The specific primer sequences utilized in all experiments can be found in Additional file 1: Table S2.

### RNase R treatment, streptolidine D treatment, and Nucleo-cytoplasmic separation

To evaluate the stability of circRNA, a treatment with RNase R was conducted. Specifically, 10 μg of total RNA extracted from specific cells was subjected to two conditions: one with RNase R (3 U/μg) and the other without, followed by an incubation period of 20 min. Additionally, Streptolidine D treatment was performed by adding 2 μg/mL of Streptolidine D (Sigma) to the designated cell culture medium. Furthermore, to investigate the distribution of RNA in different cellular locations, the cytoplasmic and nuclear RNA fractions were separated using the Cell and Nuclear RNA Purification Kit (Cat#21000, Norgen) as per the manufacturer's instructions. The relative RNA content in these distinct cellular compartments was analyzed using qPCR. For the assessment of nuclear RNA and cytoplasmic RNA separation efficiency, U6 and β-actin were utilized as reference genes, respectively. This approach enabled the study of circRNA behavior and its distribution within the cell.

### Western blot assay

To analyze protein expression in treated cells, total protein was extracted using Cell Lysis Buffer (CST) containing protease and phosphatase inhibitors for preserving protein integrity. The Bicinchoninic Acid (BCA) Protein Assay Kit (Cat#20201ES, Yeasen) was utilized to accurately quantify the protein concentration. Equal amounts of protein samples underwent Sodium Dodecyl Sulfate–Polyacrylamide Gel Electrophoresis (SDS-PAGE) for molecular weight-based separation. Subsequently, the proteins were transferred to a Polyvinylidene Fluoride (PVDF) membrane for further analysis. To minimize non-specific binding, 5% non-fat milk was used to block the membranes. Primary antibodies specific to the target proteins were incubated overnight at 4 °C, followed by incubation with secondary antibodies at room temperature for 1 h, including GAPDH (1:5000, Cat#5174, CST) and RUNX3 (1:1000, Cat#9467, CST). Enhanced Chemiluminescence (ECL) reagent (Tanon) facilitated the visualization and sensitive detection of protein bands on the membrane.

### Cell proliferation assays

The proliferation ability of PCa cells was assessed using the Cell Counting Kit-8 (Cat#A311, Vazyme), following the manufacturer's instructions with three independent replicates. In brief, 5000 cells were plated in a 96-well plate and incubated for 24 h. After that, they were treated with 10 μl CCK-8 reagent for 3 h. The absorbance was then measured at 450 nm to evaluate the cellular response.

The EdU assay (Cat#40276ES, Yeasen) was conducted to detect actively proliferating cells. 5 × 10^5^ cells were seeded in 6-well plates and cultured in DMEM supplemented with 10% FBS. When the cells reached 70% confluence, the medium was replaced with the EdU labeling mixture for 4 h. After fixation and permeabilization. the incorporation of EdU was then detected, and images were captured using a fluorescence microscope (Olympus). The data were presented as the ratio of EdU-positive cells to the total cell count, providing valuable information about cell proliferation.

### Migration assay

5 × 10^5^ cells were seeded in 6-well plates and cultured in DMEM supplemented with 10% FBS. When the cells reached full confluence, the growth medium was replaced by DMEM without FBS. After starvation for 6 h, the cells were subjected to a controlled scratch using 200 μl pipette tips. Images were taken both immediately after scratching 0 h and 24 h later. The extent of migration was determined by computing the disparity between the initial area (S0) and the area assessed at each time point (St). The migration rate was established as the migrated area divided by the initial area, expressed as a percentage: Migration rate = (S0—St)/S0 × 100%.

### Transwell assay

In the bottom layer of the well plate, 500 μl of complete culture medium was added, supplemented with 100 μl of fetal bovine serum (FBS). In the upper layer covered with Matrigel (Corning), 5 × 10^4^ cells were seeded per well without the addition of FBS. After a specific incubation period, the cells that migrated through the upper layer were fixed using 4% formalin (Beyotime). Subsequently, the migrated cells were stained with crystal violet (Beyotime) for visualization and analysis. This approach allowed for the assessment of cell migration capabilities in the context of the study.

### Fluorescence in situ hybridization (FISH)

To investigate the subcellular localization of hsa_circ_0071478 in DU145 and PC3 cells, FISH experiment was employed. For this purpose, Cy3-labeled probes specifically designed by RiboBio Co., Ltd. (Guangzhou, China) were utilized to target and capture hsa_circ_0071478. The experiment involved pre-hybridization of the cells, which were subsequently fixed on slides. Full hybridization with the hsa_circ_0071478 probe in hybridization buffer was performed to ensure accurate binding. To visualize the cell nuclei, DAPI (Yeasen) staining was employed. Finally, the slides were imaged using a confocal microscope, enabling the precise determination and visualization of the subcellular localization of hsa_circ_0071478 in the studied cell lines. The probe sequence used can be found in Additional file 1: Table S1, providing essential details about the probe used in this experiment.

### Immunofluorescence

Tissues from mice tumor embedded in paraffin wax, cut into sections, and then deparaffinized. Tissue sections were incubated in 10% goat serum and 0.5% Triton-X 100 for 30 min, and stained by Ki-67 antibody (Cat#AF7649, R&D). DAPI (1:5000, Vector Laboratories) was used to stain the cell nuclei for 30 min. Images were obtained by Olympus IX83 fluorescence microscope (Olympus Corporation, Japan).

### Luciferase reporter assay

WT-hsa_circ_0071478, Mut-hsa_circ_0071478, WT-RUNX3 and Mut-RUNX3 were designed and synthesized by Genomeditech. HEK293T cells were cultured in a 96-well plate and co-transfected with either plasmids or miR-558 mimics using Lipofectamine 2000 reagent. After 48 h, all cells were collected, and the Dual-Luciferase Reporter Assay System (Cat#E1910, Promega) was utilized to detect the interaction between miR-558 and the WT or Mut variants of hsa_circ_0071478.

### RNA-binding protein immunoprecipitation (RIP) assay

To conduct the RIP assay, the EZ-Magna RIP RNA-binding Protein Immunoprecipitation Kit (Cat#17-701, Millipore) was employed, following the manufacturer’s instructions. In brief, cells in RIP lysis buffer supplemented with protease and RNase inhibitors. The lysates were subjected to an incubation with magnetic beads that had been conjugated with AGO2 antibodies or IgG (Millipore), maintained at 4 °C overnight. Subsequently, the beads underwent a series of washes and were then treated with proteinase K to eliminate proteins. Finally, RNA was extracted according to the step in qPCR.

### RNA pulldown assay

A biotinylated-circTENM3 probe (5′-CATCTTCCTCATCACAGCAC-3′) was synthesized by RiboBio (Guangzhou, China), whereas the oligo probe (5′-TATGTTGTTGATTTGCTGGC-3′) served as a control. The circRNA pulldown procedure was conducted using the Pierce™ Magnetic RNA–Protein Pull-Down Kit (Cat#20164, Thermo Fisher Scientific).

### Differentially expressed genes selection

The publicly available Gene Expression Omnibus (GEO) dataset GSE140927 was analyzed to investigate circRNAs associated with the PCa. Differential expression genes (DEGs) analysis was performed using the R package “limma” through the GEO2R online tool (https://www.ncbi.nlm.nih.gov/geo/geo2r/). Genes with a *p*-value < 0.05 and a |log2 (Fold-change)|≥ 1 were considered significantly DEGs. The resulting DEGs were visualized using the R packages “ggplot2” for the Volcano Plot and “ComplexHeatmap” for the Heatmap.

### Animals model

All experimental procedures adhered to ethical guidelines and were approved by the relevant committees. A xenograft nude mouse model was utilized to investigate the effects of circTENM3 on tumor formation and metastatic ability in PCa. 4-week-old male BALB/c nude mice were employed and 1 × 10^6^ DU145 cells stably transfected with either the circTENM3 overexpression vector or a control vector were injected subcutaneously for the tumor formation experiment. Tumor volume was measured every 7 days and the volume was calculated as follows: volume = 4π/3 × (width/2)^2^ × (length/2).

### Statistical analysis

In this study, all data are expressed as mean ± standard deviation (SD), representing the central tendency and variability of the measurements. Statistical analysis was conducted using GraphPad Prism statistical software. The significance of differences between groups was assessed using Student's *t*-test (two-tailed) for continuous variables, and the chi-square test was employed for categorical variables. A *p*-value less than 0.05 (P < 0.05) was considered statistically significant, indicating meaningful differences between the compared groups.

## Results

### Identification and characterization of circRNAs in PCa

**Fig. 1:**
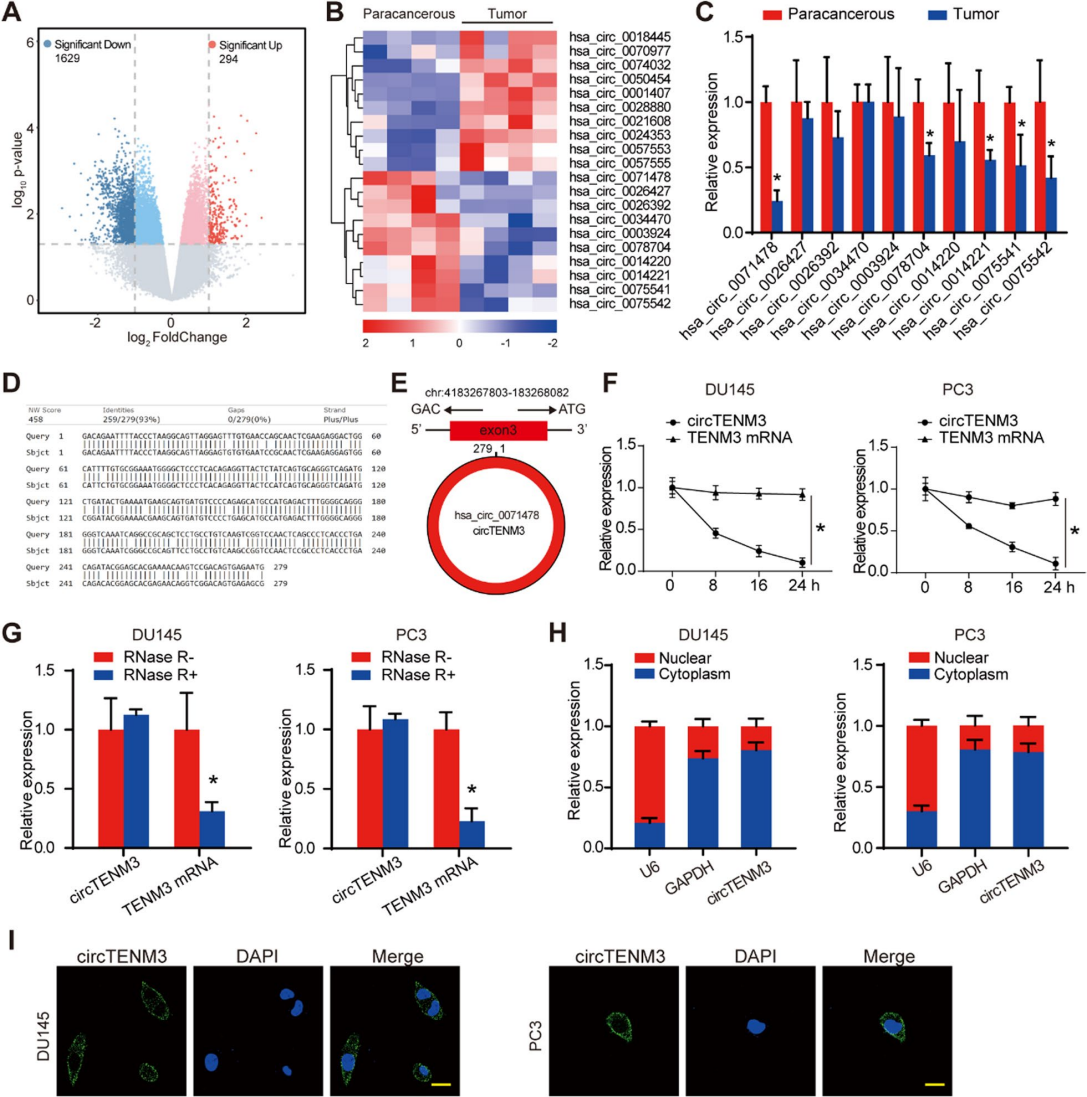
Identification and characterization of circRNAs in PCa. **A** The Volcano plot illustrated the differentially expressed circRNAs in PCa extracted from the GSE140927 dataset. **B** The cluster heatmap displayed the expression patterns of the 10 most significant upregulated and downregulated differentially expressed circRNAs. **C** The qPCR analysis examined the 10 downregulated circRNAs levels in PCa, compared to paracancerous tissue. N = 4. **D** circTENM3 homology analysis in human and mouse genome was conducted using Basic Local Alignment Search Tool. **E** Schematic presentation exhibiting the origination of circTENM3. **F** Alterations in the circTENM3 and TENM3 mRNA levels were detected by qPCR after Actinomycin D treatment in DU145 and PC3 cell lines. N = 4. **G** qPCR analysis of circTENM3 and TENM3 mRNA levels after RNase R treatment in DU145 and PC3 cell lines. N = 4. **H** The expression of nuclear control transcript (U6), cytoplasm control transcript (GAPDH) and circTENM3 was detected by qPCR in the nuclear and cytoplasm fractions of DU145 and PC3 cell lines. N = 4. **I** RNA-FISH assays were conducted to detect circTENM3 expression in DU145 and PC3 cell lines using anti-circTENM3 probes. Nuclei were stained with 4′,6-diamidino-2-phenylindole (DAPI). Scale bar = 20 μm. All data were presented as the mean ± SD. C, G, unpaired Student’s *t*-test. F, Two-way ANOVA with Bonferroni post hoc test. *P < 0.05

We conducted a study to identify circRNAs that may play a role in the progression of PCa. For this purpose, we screened the GEO database (GSE140927) to find potential circRNAs involved in PCa development. Our analysis revealed 1629 downregulated and 294 upregulated genes in PCa compared to paracancerous tissue (Fig. 1a). We further investigated the ten most significant downregulated and upregulated genes through heatmap clustering analysis (Fig. 1b). After validating the downregulated genes by qPCR analysis, we focused on hsa_circ_0071478, which exhibited the most significant downregulation among PCa tissues (Fig. 1c). This circRNA is highly conserved in the human genome from chr4: 4,183,267,803 to 183,268,082 and in the mouse genome from ch8: 49,731,611 to 49,731,890 (mmu_circ_0014855) (Fig. 1d). Due to its host gene being TENM3, we named it circTENM3 for functional characterization (Fig. 1e).

To investigate the stability of circTENM3, we treated DU145 and PC3 cell lines with the transcription inhibitor Actinomycin D. The experimental results demonstrated that circTENM3 exhibited higher stability compared to TENM3 mRNA (Fig. 1f). Additionally, circTENM3 showed resistance to RNase R treatment, in contrast to TENM3 mRNA, which experienced significant degradation (Fig. 1g). Cellular localization experiments using qPCR and FISH confirmed that circTENM3 predominantly localized in the cytoplasm, not in the nucleus (Fig. 1h, i). These findings indicate the stable nature of circTENM3 and its cytoplasmic distribution in the studied cell lines.

### CircTENM3 inhibits PCa progression in vitro

**Fig. 2:**
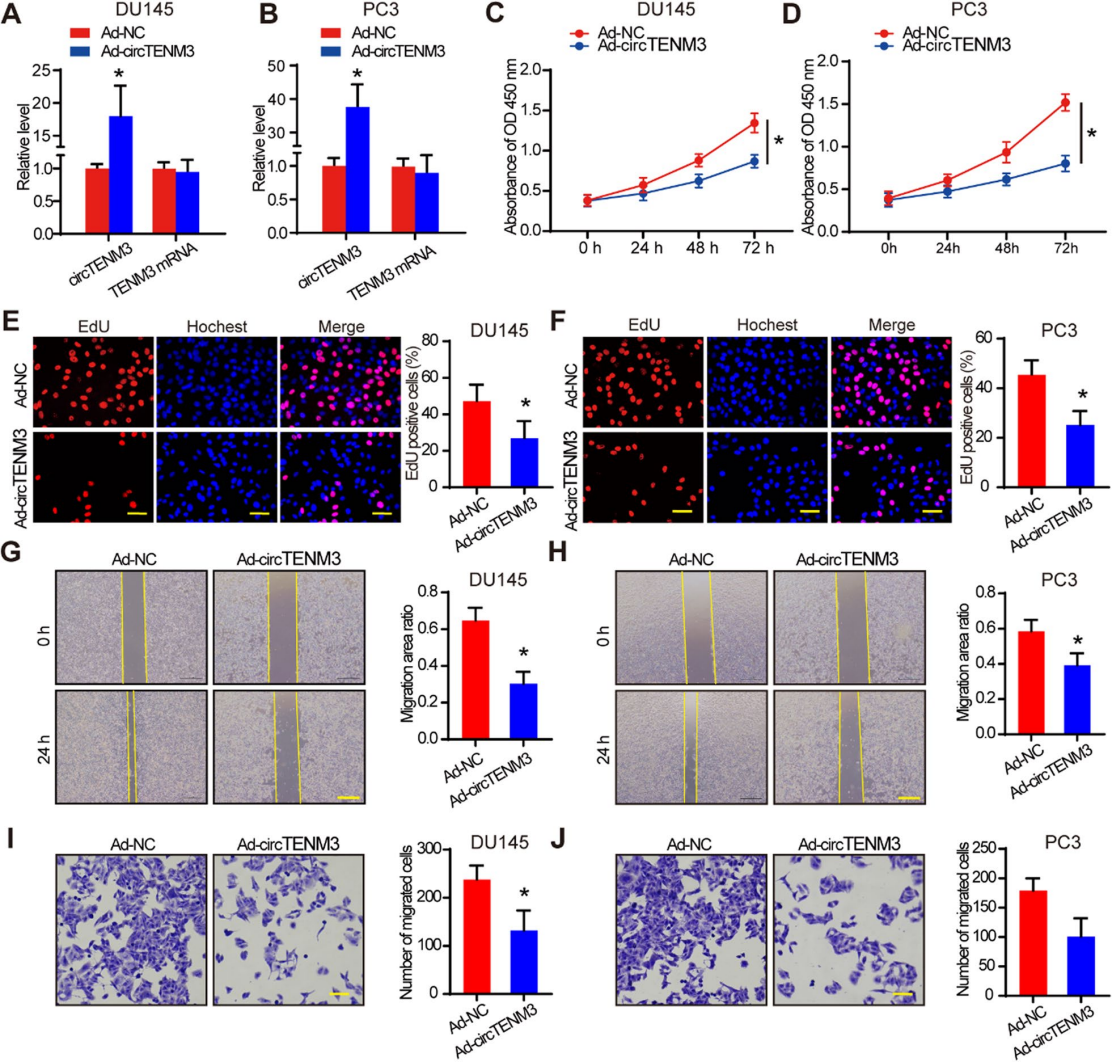
CircTENM3 inhibits PCa progression in vitro. **A**, **B** qPCR was used to determine the efficacy of circTENM3 overexpression in DU145 and PC3 cells, compared to the negative control (Ad-NC). N = 4. **C**, **D**, CCK-8 assays were performed in DU145 and PC3 cell with circTENM3 overexpression. N = 4. **E**, **F**, EdU assays were performed in DU145 and PC3 cell with circTENM3 overexpression. N = 4. Scale bar = 50 μm. **G**, **H**, Scratch assays were performed in DU145 and PC3 cell with circTENM3 overexpression. N = 4. Scale bar = 200 μm. **I**, **J**, Transwell assays were performed in DU145 and PC3 cell with circTENM3 overexpression. N = 4. Scale bar = 50 μm. All data were presented as the mean ± SD. **A**, **B**, **E**, **F**, **G**, **H**, **I**, **J** unpaired Student’s *t*-test. **C**, **D**, Two-way ANOVA with Bonferroni post hoc test. *P < 0.05

We conducted various in vitro experiments to explore the effect of circTENM3 on PCa cells. By constructing an adenovirus expressing circTENM3, we achieved efficient circTENM3 overexpression in PC3 cells without affecting TENM3 mRNA levels (Fig. 2a, b). Subsequently, we observed that circTENM3 overexpression significantly suppressed the growth of DU145 and PC3 cell lines (Fig. 2c, d). EdU assay confirmed the inhibitory effect of circTENM3 overexpression on cell proliferation in DU145 and PC3 cell lines (Fig. 2e, f). Moreover, overexpression of circTENM3 inhibited cell migration (Fig. 2g, h) and invasion (Fig. 2i, j) in both DU145 and PC3 cell lines. These results indicated that circTENM3 negatively regulated PCa progression in vitro.

### CircTENM3 acts as a miR-558 sponge

**Fig. 3:**
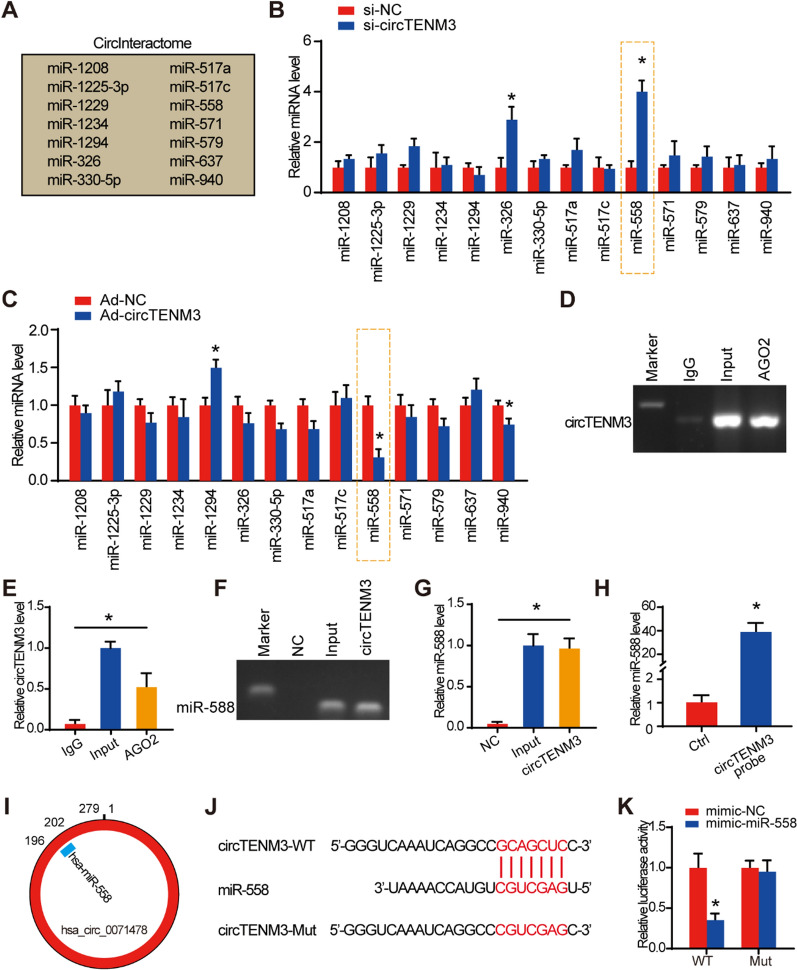
CircTENM3 acts as a miR-558 sponge. **A** CircInteractome database showed the predicted downstream targets of circTENM3. **B** qPCR analysis was used to determine the effect of circTENM3 kncokdown on the predicted downstream targets in DU145 cells. N = 4. **C** qPCR analysis was used to determine the effect of circTENM3 overexpression on the predicted downstream targets in DU145 cells. N = 4. **D**–**G** The association between circTENM3, miR-558 and AGO2 was ascertained by analyzing DU145 cell lysates using RNA immunoprecipitation with an AGO2 antibody. qPCR was used to detect the change of circTENM3 (**D**, **E**) and miR-558 (**F**, **G**) levels. N = 4. **H**, DU145 cell lysis was pulled down and enriched with circTENM3 specific probe and then detected the expression of miR-558 by qPCR. N = 4. **I**, The binding site of circTENM3 and miR-558. **J**, The direct binding sites between circTENM3 and miR-558. **K**, Luciferase reporter assay was performed to confirm the direct binding relationship between circTENM3 and miR-558. N = 4. All data were presented as the mean ± SD. **B**, **C**, **H**, **K**, unpaired Student’s *t*-test. **E**, **G**, One-way ANOVA with Bonferroni post hoc test. *P < 0.05

Based on accumulating evidence revealing the regulatory roles of circRNAs as miRNA sponges in PCa [22, 23], we explored the potential function of circTENM3 in tumor progression due to its specific subcellular localization and exceptional stability. To investigate the underlying molecular mechanism, we employed the CircInteractome database and identified 14 candidate miRNAs that could potentially interact with circTENM3 (Fig. 3a). Further experiments demonstrated that knockdown of circTENM3 in DU145 cell line led to a significant increase in the expression of two miRNAs, miR-326 and miR-558, with miR-558 showing the highest upregulation (Fig. 3b). Conversely, overexpression of circTENM3 markedly decreased the expression levels of miR-558 and miR-940 (Fig. 3c). These findings conjointly counsel that miR-558 is that the essential miRNA related to circTENM3 in PCa cells.

To ascertain the functional role of circTENM3 as a molecular sponge for miR-558, we conducted a RIP assay utilizing an AGO2 antibody. The obtained results demonstrated the presence of circTENM3 and miR-558 in the immunoprecipitate pulled down by the AGO2 antibody, thereby indicating its specific interaction with miRNAs through AGO2 (Fig. 3d–g). Additionally, a pull-down assay was conducted using a biotin-coupled circTENM3 probe to assess the binding of circTENM3 to miR-558. The biotin-coupled circTENM3 probe group exhibited a significant enrichment of miR-558 compared to the negative control group (Fig. 3h), further supporting the potential binding between circTENM3 and miR-558 (Fig. 3i). To assess the functional downregulation of miR-558 by circTENM3, luciferase reporter gene experiments were conducted. The luciferase reporter assay revealed a significant reduction in reporter activity in the wild-type group of circTENM3, in contrast to the mutant type group (Fig. 3j, k). These results conclusively demonstrate the direct binding of circTENM3 to miR-558 in PCa cells, establishing its role as a molecular sponge in regulating miR-558’s function.

### CircTENM3 reverses the effects of miR-558 in PCa in vitro

**Fig. 4:**
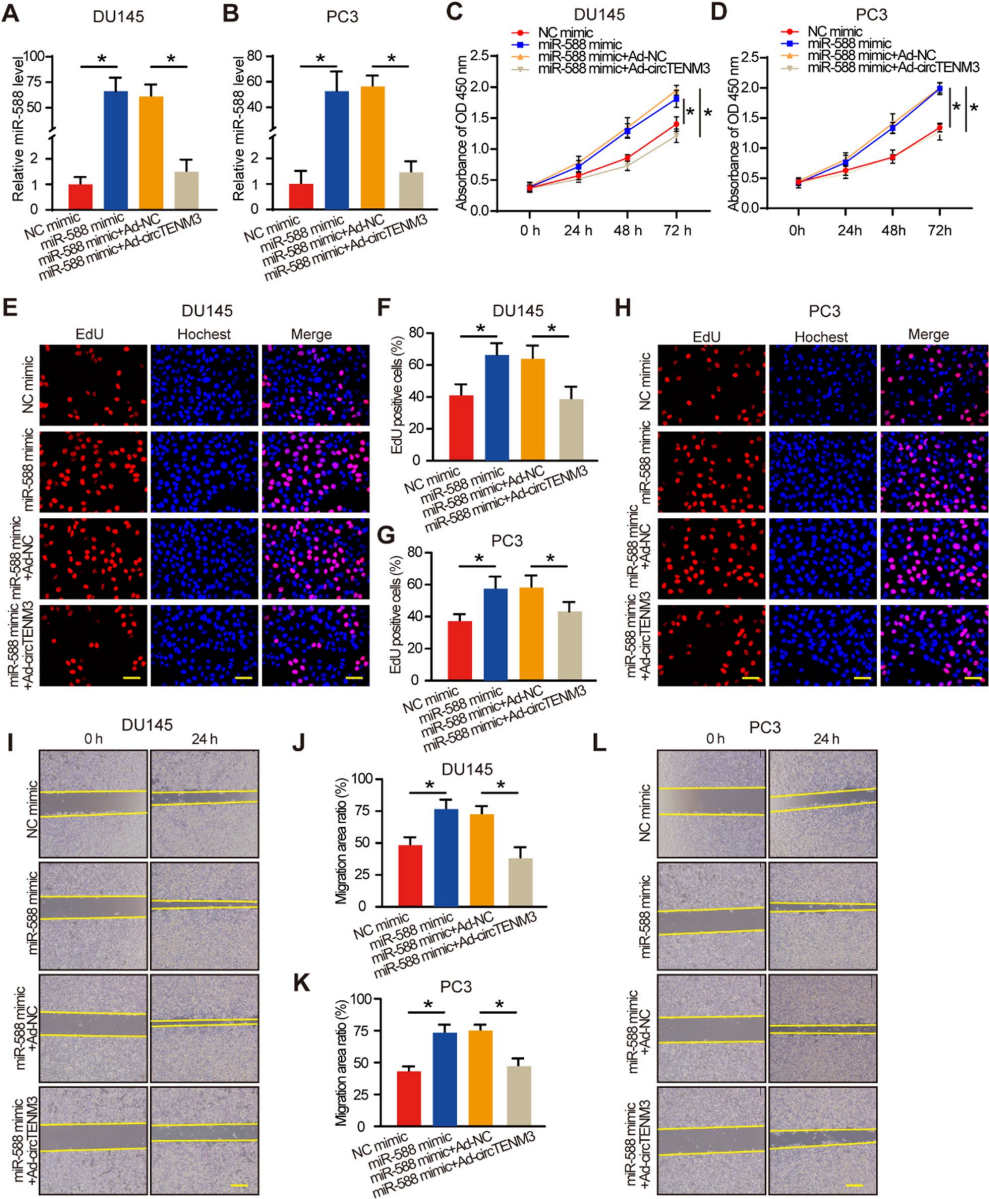
CircTENM3 reverses the effects of miR-558 in PCa. **A**, **B** qPCR was used to determine the efficacy of miR-558 mimic with or without circTENM3 overexpression in DU145 and PC3 cells. N = 4. **C**, **D** CCK-8 assays were performed in DU145 and PC3 cell to detect the effect of miR-558 with or without circTENM3 overexpression. N = 4. **E**–**H** EdU assays were performed in DU145 and PC3 cell to detect the effect of miR-558 with or without circTENM3 overexpression. N = 4. Scale bar = 50 μm. **I**–**L** Scratch assays were performed in DU145 and PC3 cell to detect the effect of miR-558 with or without circTENM3 overexpression. N = 4. Scale bar = 200 μm. All data were presented as the mean ± SD. **A**, **B**, **C**, **D**, **F**, **G**, **J**, **K**, Two-way ANOVA with Bonferroni post hoc test. *P < 0.05

To experimentally validate the functional role of circTENM3 as a miR-558 sponge in inhibiting PCa progression, we conducted rescue experiments utilizing miR-558 mimic and circTENM3 adenovirus. Effective regulation of miR-558 levels was achieved by transfecting miR-558 mimics and the expression levels of miR-558 were reversed by circTENM3 adenovirus both in DU145 cells (Fig. 4a) and PC3 cells (Fig. 4b). CCK-8 assay demonstrated that miR-558 mimic inhibited the proliferation of PCa cell lines and the overexpression of circTENM3 reversed the inhibition of miR-558 (Fig. 4c, d). Additionally, the EdU assay further confirmed that circTENM3 reversed the promoting effects of miR-558 on cell proliferation (Fig. 4e–h). Furthermore, in both DU145 and PC3 cell lines, the migration (Fig. 4i–l) promotion induced by miR-558 were alleviated by overexpression of circTENM3. These findings collectively support the notion that circTENM3 functions as a miR-558 sponge, contributing to the inhibition of PCa progression.

### CircTENM3 enhances RUNX3 levels via miR-558 sponge

**Fig. 5:**
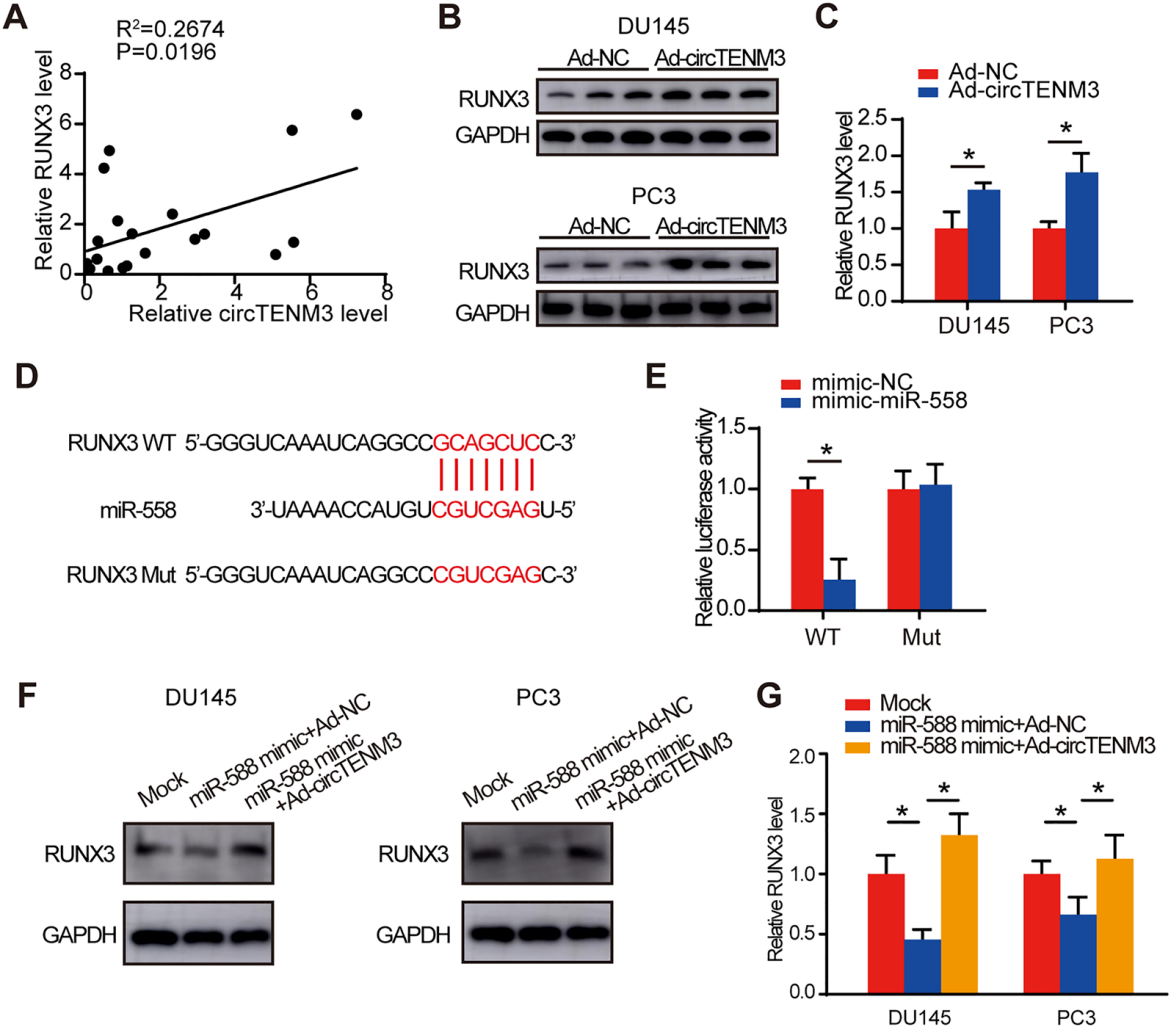
CircTENM3 enhances RUNX3 levels via miR-558 sponge. **A** The correlation between circTENM3 and miR-558 in PCa. N = 20. **B**, **C** Western blot assay was performed to assess the RUNX3 protein levels in DU145 and PC3 cells with circTENM3 overexpression. N = 3. **D** The direct binding sites between miR-558 and RUNX3. **E** Luciferase reporter assay was performed to confirm the direct binding relationship between miR-558 and RUNX3. N = 4. **F**, **G** Western blot assay was performed to assess the effect of miR-558 on RUNX3 protein levels in DU145 and PC3 cells with or without circTENM3 overexpression. N = 3. All data were presented as the mean ± SD. **C**, **E**, unpaired Student’s *t*-test. **G** One-way ANOVA with Bonferroni post hoc test. *P < 0.05

According to the sequences predicted from the Targetscan database, both RUNX3 and circTENM3 were identified to target the same element of miR-558. Previous studies have indicated that miR-558 has the capability to target RUNX3 [24]. To investigate the relationship between circTENM3 and RUNX3, qPCR was used to quantified the relative levels of circTENM3 and RUNX3 in PCa tissues. Interestingly, the expression levels of RUNX3 showed a positive correlation with the circTENM3 (Fig. 5a). Next, the effect of circTENM3 on RUNX3 was further investigated in PCa cells. The results indicated that RUNX3 protein levels in PCa cells were decreased with the overexpression of circTENM3 (Fig. 5b, c), further supporting the inverse relationship between circTENM3 and RUNX3.

To validate the interaction between miR-558 and RUNX3, we performed a luciferase reporter assay in HEK293T cells. These cells were transfected with either the wild-type or mutant RUNX3 sequence (Fig. 5d). The results from the luciferase reporter assay revealed a significant reduction in cells transfected with the wild-type RUNX3, as compared to cells with the mutant sequence (Fig. 5e). This finding provides additional evidence supporting the direct binding between miR-558 and RUNX3. Additionally, DU145 and PC3 cell lines, which exhibited either overexpression of circTENM3 or negative control, were transfected with miR-558 mimics. The outcomes of this experiment demonstrated that the circTENM3-induced alterations in the levels of RUNX3 protein in PCa cells (Fig. 5f, g) were effectively counteracted by the application of miR-558 inhibitors and mimics, respectively. These observations serve to further corroborate the functional interplay existing among circTENM3, miR-558, and RUNX3 within the context of PCa cells.

### CircTENM3 suppresses PCa progression in vivo

**Fig. 6:**
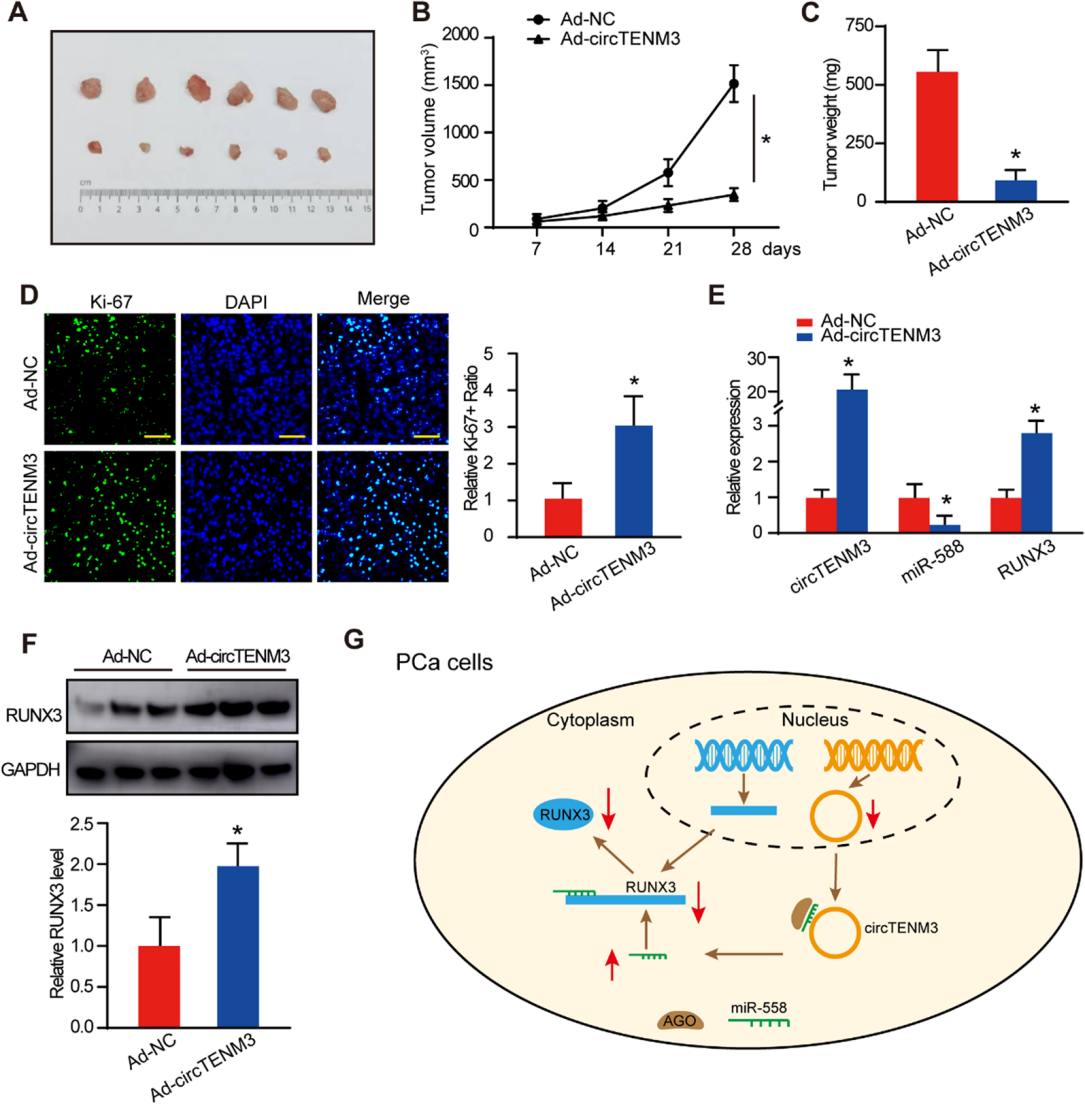
CircTENM3 suppresses PCa progression in vivo. **A** Photographs of tumors obtained from the Ad-NC and Ad-circTENM3 groups of nude mice. N = 6. **B** Tumor volumes were measured once a week after tumor formation. **C** Tumors were removed and weighed 28 days after implantation. **D** Immunofluorescence staining of Ki-67 in the tumors obtained from the Ad-NC and Ad-circTENM3 groups of nude mice. N = 6. **E** qPCR was performed to assess the relative circTENM3, miR-558 and RUNX3. **F** RUNX3 protein levels of the Ad-NC and Ad-circTENM3 groups. N = 3. **G** Schematic illustration presented the molecular mechanism of circTENM3 in PCa cells. The downregulation of circTENM3 leaded to an increase of miR558 and the decrease of RUNX3, which promoted the proliferation of PCa cells. All data were presented as the mean ± SD. **B** Two-way ANOVA with Bonferroni post hoc test. **C**, **D**, **E**, **F** unpaired Student’s *t*-test. *P < 0.05

To gain deeper insights into the in vivo implications of circTENM3 in PCa, we conducted subcutaneous inoculation of DU145 cells expressing either Ad-NC or Ad-circTENM3 into BALB/c xenograft mouse models. Strikingly, our results underscored that the overexpression of circTENM3 markedly impeded PCa tumor growth in the in vivo setting (Fig. 6a). The temporal analysis of PCa tumor growth demonstrated that, after 28 days, the Ad-circTENM3 group exhibited significantly smaller tumor volumes (Fig. 6b) and reduced tumor weight (Fig. 6c) in comparison to the Ad-NC group. Moreover, we quantified the relative levels of circTENM3, RUNX3, and miR-558 in tumor tissue specimens using qPCR (Fig. 6d). Remarkably, the Ad-circTENM3 group displayed elevated levels of both circTENM3 and RUNX3, while miR-558 exhibited a contrary trend, in accordance with the in vitro experimental results. Additionally, Western blot analysis validated the upregulation of the RUNX3 protein level upon circTENM3 overexpression (Fig. 6e, f). In conclusion, the presented findings provide compelling evidence that circTENM3 exerts an inhibitory effect on the growth of PCa, which aligns with the observations made in the in vitro experiments.

## Discussion

The exploration and comprehension of novel covalently closed circular transcripts and their functions have been significantly advanced by cutting-edge sequencing and specialized bioinformatics methods [25, 26]. Recent research has shed light on the pivotal role played by circRNAs in diverse cellular and physiological processes, particularly in the context of PCa progression [27–29]. Despite these valuable findings, there still remains much to be explored. In our study, we found that circTENM3 was downregulated in PCa. CircTENM3 was derived from exon 3 of the TENM3 gene, and its function was unknown. Through functional experiments, it has been demonstrated that overexpression of circTENM3 in PCa cells suppresses their proliferation, migration, and invasion abilities in vitro, as well as inhibits tumor growth in vivo. This discovery suggested that circTENM3 had the potential to serve as a new and promising biomarker and inhibitory molecule in PCa cells.

CircRNAs play a pivotal role in modulating cancer development through diverse mechanisms, including miRNA sponging, peptide encoding, protein interaction, and Wang et al. [30] and Patop et al. [33]. The intriguing ability of cytoplasmic circRNAs to function as miRNA sponges, post-transcriptionally inhibiting miRNAs from targeting downstream genes, significantly impacts tumor progression [34–36]. In the case of circTENM3, which originated from TENM3 encoding exons and predominantly localizes in the cytoplasm, we postulated that it might act as a miRNA sponge, thereby regulating downstream target genes. So RIP, FISH, luciferase reporter gene assay, and RNA pulldown analysis were used to reveal that circTENM3 seved as a sponge for miR-558 in PCa cells. Additionally, the rescue experiments revealed that the upregulation of miR-558 in PCa cells counteracted the anticancer effects of circTENM3. Bioinformatics revealed that RUNX3 was the downstream target gene of miR-558, which was further confirmed by western blot and luciferase reporter assay. These findings suggested that circTENM3 functioned as a miR-558 sponge and regulated the progression of PCa cells by indirectly modulating RUNX3.

In the realm of nuclear transcriptional regulators, RUNX3 holds a distinguished position as a significant member of the Runt domain family [37]. Its contributions span a diverse array of biological processes, playing pivotal roles in crucial activities such as development, cell proliferation, differentiation, senescence, DNA repair, and inflammation [38]. Notably, its paramount importance stems from its firmly established reputation as a potent tumor suppressor. This becomes even more noteworthy due to its consistent observation of inactivation across a spectrum of cancer types, ranging from gastric and lung to breast cancers [39, 40]. RUNX3 had the ability to form a ternary complex with beta-catenin/TCF4, leading to the suppression of Wnt signaling activity and the suppression of tumor [41]. Our study revealed that circTENM3 could repressed the progression of PCa via the expression of RUNX3 levels.

In conclusion, our study revealed a notable decrease of circTENM3 expression in PCa tissues. Mechanistically, circTENM3 suppressed the proliferation and invasion of PCa cells by acting as a miR-558 sponge and upregulating RUNX3 expression. These findings suggested that circTENM3 could be a promising biomarker of PCa and circTENM3 vaccines might be a potential therapy for patients with PCa, which set the direction for our future advancements.

## Conclusion

In conclusion, our study provides deep insights into the protective function of circTENM3 in PCa. Mechanistically, circTENM3 functions as a miR-558 sponge, leading to the upregulation of RUNX3 and subsequent inhibition of PCa progression. This highlights the potential significance of therapeutic approaches targeting the circTENM3/miR-558/RUNX3 axis for effective PCa treatment.

### Supplementary Information

**Additional file 1. Table S1**: Sequence of siRNAs and mimics. **Table S2**: Sequence of primers.

## Data Availability

All data generated or analysed during this study are included in this published article.

## Abbreviations

PCa: Prostate cancer
circRNA: Circular RNA
miRNA: MicroRNA
siRNA: Small interfering RNA
qPCR: Quantitative polymerase chain reaction
FISH: Fluorescence in situ hybridization
RIP: RNA-binding protein immunoprecipitation

## References

[1] Bergengren O, Pekala KR, Matsoukas K, Fainberg J, Mungovan SF, Bratt O, Bray F, Brawley O, Luckenbaugh AN, Mucci L, Morgan TM, Carlsson SV (2023). 2022 update on prostate cancer epidemiology and risk factors—a systematic review. Eur Urol.

[2] Siegel RL, Miller KD, Jemal A (2020). Cancer statistics, 2020. CA Cancer J Clin.

[3] Malik JA, Ahmed S, Momin SS, Shaikh S, Alafnan A, Alanazi J, Said Almermesh MH, Anwar S (2023). Drug repurposing: a new hope in drug discovery for prostate cancer. ACS Omega.

[4] Rebello RJ, Oing C, Knudsen KE, Loeb S, Johnson DC, Reiter RE, Gillessen S, Van der Kwast T, Bristow RG (2021). Prostate cancer. Nat Rev Dis Primers.

[5] Kohaar I, Petrovics G, Srivastava S (2019). A rich array of prostate cancer molecular biomarkers: opportunities and challenges. Int J Mol Sci.

[6] Adamaki M, Zoumpourlis V (2021). Prostate cancer biomarkers: from diagnosis to prognosis and precision-guided therapeutics. Pharmacol Ther.

[7] Kristensen LS, Andersen MS, Stagsted LVW, Ebbesen KK, Hansen TB, Kjems J (2019). The biogenesis, biology and characterization of circular RNAs. Nat Rev Genet.

[8] Li X, Yang L, Chen LL (2018). The biogenesis, functions, and challenges of circular RNAs. Mol Cell.

[9] Kristensen LS, Jakobsen T, Hager H, Kjems J (2022). The emerging roles of circRNAs in cancer and oncology. Nat Rev Clin Oncol.

[10] Abe BT, Wesselhoeft RA, Chen R, Anderson DG, Chang HY (2022). Circular RNA migration in agarose gel electrophoresis. Mol Cell.

[11] Guria A, Sharma P, Natesan S, Pandi G (2019). Circular RNAs-the road less traveled. Front Mol Biosci.

[12] Chen LL, Bindereif A, Bozzoni I, Chang HY, Matera AG, Gorospe M, Hansen TB, Kjems J, Ma XK, Pek JW, Rajewsky N, Salzman J, Wilusz JE, Yang L, Zhao F (2023). A guide to naming eukaryotic circular RNAs. Nat Cell Biol.

[13] Chen L, Shan G (2021). CircRNA in cancer: fundamental mechanism and clinical potential. Cancer Lett.

[14] Yu T, Wang Y, Fan Y, Fang N, Wang T, Xu T, Shu Y (2019). CircRNAs in cancer metabolism: a review. J Hematol Oncol.

[15] Lei M, Zheng G, Ning Q, Zheng J, Dong D (2020). Translation and functional roles of circular RNAs in human cancer. Mol Cancer.

[16] Hong W, Xue M, Jiang J, Zhang Y, Gao X (2020). Circular RNA circ-CPA4/let-7 miRNA/PD-L1 axis regulates cell growth, stemness, drug resistance and immune evasion in non-small cell lung cancer (NSCLC). J Exp Clin Cancer Res CR.

[17] Zhou WY, Cai ZR, Liu J, Wang DS, Ju HQ, Xu RH (2020). Circular RNA: metabolism, functions and interactions with proteins. Mol Cancer.

[18] Wu P, Mo Y, Peng M, Tang T, Zhong Y, Deng X, Xiong F, Guo C, Wu X, Li Y, Li X, Li G, Zeng Z, Xiong W (2020). Emerging role of tumor-related functional peptides encoded by lncRNA and circRNA. Mol Cancer.

[19] Yu YZ, Lv DJ, Wang C, Song XL, Xie T, Wang T, Li ZM, Guo JD, Fu DJ, Li KJ, Wu DL, Chan FL, Feng NH, Chen ZS, Zhao SC (2022). Hsa_circ_0003258 promotes prostate cancer metastasis by complexing with IGF2BP3 and sponging miR-653-5p. Mol Cancer.

[20] Xie T, Fu DJ, Li ZM, Lv DJ, Song XL, Yu YZ, Wang C, Li KJ, Zhai B, Wu J, Feng NH, Zhao SC (2022). CircSMARCC1 facilitates tumor progression by disrupting the crosstalk between prostate cancer cells and tumor-associated macrophages via miR-1322/CCL20/CCR6 signaling. Mol Cancer.

[21] Ding L, Wang R, Zheng Q, Shen D, Wang H, Lu Z, Luo W, Xie H, Ren L, Jiang M, Yu C, Zhou Z, Lin Y, Lu H, Xue D, Su W, Xia L, Neuhaus J, Cheng S, Li G (2022). circPDE5A regulates prostate cancer metastasis via controlling WTAP-dependent N6-methyladenisine methylation of EIF3C mRNA. J Exp Clin Cancer Res CR.

[22] Hansen TB, Jensen TI, Clausen BH, Bramsen JB, Finsen B, Damgaard CK, Kjems J (2013). Natural RNA circles function as efficient microRNA sponges. Nature.

[23] Li J, Sun D, Pu W, Wang J, Peng Y (2020). Circular RNAs in cancer: biogenesis, function, and clinical significance. Trends Cancer.

[24] Guo X, Dai X, Liu J, Cheng A, Qin C, Wang Z (2020). Circular RNA circREPS2 acts as a sponge of miR-558 to suppress gastric cancer progression by regulating RUNX3/β-catenin signaling. Mol Therapy Nucl Acids.

[25] Vo JN, Cieslik M, Zhang Y, Shukla S, Xiao L, Zhang Y, Wu YM, Dhanasekaran SM, Engelke CG, Cao X, Robinson DR, Nesvizhskii AI, Chinnaiyan AM (2019). The landscape of circular RNA in cancer. Cell.

[26] Liu CX, Chen LL (2022). Circular RNAs: characterization, cellular roles, and applications. Cell.

[27] Chen S, Huang V, Xu X, Livingstone J, Soares F, Jeon J, Zeng Y, Hua JT, Petricca J, Guo H, Wang M, Yousif F, Zhang Y, Donmez N, Ahmed M, Volik S, Lapuk A, Chua MLK, Heisler LE, Foucal A, Fox NS, Fraser M, Bhandari V, Shiah YJ, Guan J, Li J, Orain M, Picard V, Hovington H, Bergeron A, Lacombe L, Fradet Y, Têtu B, Liu S, Feng F, Wu X, Shao YW, Komor MA, Sahinalp C, Collins C, Hoogstrate Y, de Jong M, Fijneman RJA, Fei T, Jenster G, van der Kwast T, Bristow RG, Boutros PC, He HH (2019). Widespread and functional RNA circularization in localized prostate cancer. Cell.

[28] Zhang ZH, Wang Y, Zhang Y, Zheng SF, Feng T, Tian X, Abudurexiti M, Wang ZD, Zhu WK, Su JQ, Zhang HL, Shi GH, Wang ZL, Cao DL, Ye DW (2023). The function and mechanisms of action of circular RNAs in Urologic Cancer. Mol Cancer.

[29] Hua JT, Chen S, He HH (2019). Landscape of noncoding RNA in prostate cancer. Trends Genet TIG.

[30] Wang J, Zhu S, Meng N, He Y, Lu R, Yan GR (2019). ncRNA-encoded peptides or proteins and cancer. Mol Ther.

[31] Yang L, Wilusz JE, Chen LL (2022). Biogenesis and regulatory roles of circular RNAs. Annu Rev Cell Dev Biol.

[32] Huang A, Zheng H, Wu Z, Chen M, Huang Y (2020). Circular RNA–protein interactions: functions, mechanisms, and identification. Theranostics.

[33] Patop IL, Wüst S, Kadener S (2019). Past, present, and future of circRNAs. Embo J.

[34] Zhang C, Wang S, Chao F, Jia G, Ye X, Han D, Wei Z, Liu J, Xu G, Chen G (2023). The short inverted repeats-induced circEXOC6B inhibits prostate cancer metastasis by enhancing the binding of RBMS1 and HuR. Mol Ther.

[35] Chao F, Song Z, Wang S, Ma Z, Zhuo Z, Meng T, Xu G, Chen G (2021). Novel circular RNA circSOBP governs amoeboid migration through the regulation of the miR-141-3p/MYPT1/p-MLC2 axis in prostate cancer. Clin Transl Med.

[36] Qi JC, Yang Z, Lin T, Ma L, Wang YX, Zhang Y, Gao CC, Liu KL, Li W, Zhao AN, Shi B, Zhang H, Wang DD, Wang XL, Wen JK, Qu CB (2021). CDK13 upregulation-induced formation of the positive feedback loop among circCDK13, miR-212-5p/miR-449a and E2F5 contributes to prostate carcinogenesis. J Exp Clin Cancer Res CR.

[37] Ito Y (2004). Oncogenic potential of the RUNX gene family: 'overview'. Oncogene.

[38] Ito Y (2008). RUNX genes in development and cancer: regulation of viral gene expression and the discovery of RUNX family genes. Adv Cancer Res.

[39] Lin FC, Liu YP, Lai CH, Shan YS, Cheng HC, Hsu PI, Lee CH, Lee YC, Wang HY, Wang CH, Cheng JQ, Hsiao M, Lu PJ (2012). RUNX3-mediated transcriptional inhibition of Akt suppresses tumorigenesis of human gastric cancer cells. Oncogene.

[40] Chen LF (2012). Tumor suppressor function of RUNX3 in breast cancer. J Cell Biochem.

[41] Ito K, Lim AC, Salto-Tellez M, Motoda L, Osato M, Chuang LS, Lee CW, Voon DC, Koo JK, Wang H, Fukamachi H, Ito Y (2008). RUNX3 attenuates beta-catenin/T cell factors in intestinal tumorigenesis. Cancer Cell.

